# Platelet rich plasma versus glucocorticoid for plantar fasciitis: A protocol for systematic review and meta-analysis of randomized controlled trials

**DOI:** 10.1097/MD.0000000000027810

**Published:** 2021-12-03

**Authors:** Rongrong Ge, Shuying Chen, Jiawang Jiang, Bangmin Wang, You Zeng, Qianan Cao, Jun Wu, Yunfeng Liu

**Affiliations:** Jiangxi University of Traditional Chinese Medicine, Nanchang, China.

**Keywords:** glucocorticoid, meta-analysis, plantar fasciitis, platelet rich plasma, protocol, systematic review

## Abstract

**Background:**

Plantar fasciitis (PF) is the most common cause of heel pain in adult. There are a variety of ways to treat PF, but these treatments have varied result in their effectiveness, and exist different degrees of limitations. At present, clinical studies focus on the effect of glucocorticoid (GC) and platelet rich plasma (PRP) in the treatment of PF, but there is a lack of systematic evaluation PRP and GC's clinical effect towards PF. This study aims to evaluate the long-term efficacy of GCs and PRP in the treatment of PF by means of meta-analysis.

**Methods:**

The literature of a randomized controlled clinical trial of PRP in the treatment of plantantifasciitis was searched on the Internet. Retrieve 7 databases. EndNote X9 software was used for document management. The Jadad scale was used to score the literature. Risk assessment of the literature was conducted according to Cochrane's systematic evaluation manual 5.0. RevMan5.3 software was used for literature risk bias analysis. Stata12.0 software is used for sensitivity analysis.

**Results:**

This study will provide effective evidence-based evidence for the long-term efficacy of PRP and GC in treating PF.

**Conclusion:**

A systematic review and meta-analysis were conducted for the comparison of the long-term effect of PRP and GC on plantar fascia in the treatment of PF.

## 1 Introduction

Plantar fasciitis (PF) is a self-limited disease characterized by pain in the medial plantar after getting up or prolonged sitting along with mild plantar flexion and varus in the foot during walking and localized tenderness in the medial side of calcaneal tuberosity.^[1,2]^ PF is the most common cause of chronic heel pain in adults.^[3]^ The pathological mechanism of PF is unclear, but it has been widely accepted for medical workers that PF is caused by heavy load or excessive tension results in a slight tear in the fascia. And under continuous micro tears and chronic damage accumulation, the chronic inflammation of the fascia is building up.^[4]^ Also, some scholars believe that the emergence of the disease is closely related to obesity, standing up for a long time, incorrect walking posture, unfit shoes, the habit of work and study, and other factors.^[5,6]^ According to epidemiological studies, the incidence of PF in the United States is about 10%,^[7]^ and it is estimated that PF accounts for 11% to 15% of all foot diseases.^[8]^ Besides, studies have found that most PF patients are aged between 25 and 64 years old, with the highest incidence between 45 and 64.^[9]^ Moreover, the incidence rate of athletes or regular runners and soldiers is significantly higher than that of the general population, which is about 4% to 22%.^[10]^

At present, the treatment of PF focuses on pain relief, and mainly adopt conservative treatment. Common treatment measures include having a rest, icing plantar locally, using non-steroidal anti-inflammatory drugs, adopting physical therapy, using foot orthoses, injecting corticosteroid, etc.^[1,11]^ However, although there are a variety of ways to treat PF, these treatments have varied result in their effectiveness, and exist different degrees of limitations.^[12]^ Therefore, it is very important to find effective treatment for PF.

The research shows that PF is a kind of local inflammation triggered by abnormal tension of both abnormalities of foot and fascia plantaris.^[13]^ The pathological manifestation turns to be rupture of fascia fiber and local aseptic inflammation. The common treatment in clinical is to inject glucocorticoid (GC) into the specific point on fascia.^[14]^ GC does show a distinguished inhibitory effects when it is up to physiological level. But in the late stage of GC treatment, it easily causes rupture of metatarsal fascia and atrophia of plantar fat pad.^[15]^ Focus on this question, the clinical discovered platelet rich plasma (PRP) promotes the proliferation of bone marrow mesenchymal stem cell, adipose mesenchymal stem cell, and tendon cell in plantar fascia,^[16]^ and accelerates tendon's repair.^[17]^ Which can be used in PF's treatment. Whereas, although there have been some randomized controlled trials (RCTs) of PRP and GC in the treatment of PF, there is a lack of systematic evaluation PRP and GC's clinical effect towards PF. To fulfill it, this study was conducted by meta-analysis, to compare the long-term effect between PRP and GC towards PF. Wish to provide evidence for clinical practice.

## 2 Methods

### 2.1 Protocol registration

The protocol study has been registered on the Inplasy website (registration number is INPLASY2021100067: https://inplasy.com/inplasy-2021-10-0067/), and the systematic review protocol of us will be performed strictly following the guidelines of the Preferred Reporting Items for Systematic Review and Meta-Analysis Protocols (PRISMA-P) for systematic evaluation and meta-analysis.^[18]^

### 2.2 Source of literature and search strategy

CBMdisc, the Wanfang Chinese digital periodical and conference database, China National Knowledge Infrastructure database, the VIP Chinese Science and Technique Journals Database, the Cochrane Library, PubMed, and EMBASE were been searched from their inception to October 10, 2019. And the English terms was: “Plantar Fasciitis, Policeman's Heel, Heel, Policeman's, Heels, Policeman's, Policeman Heel, Policeman's Heels, Policemans Heel, Heel Spur Syndrome, Chronic Plantar Fasciitis, Fasciitis, Chronic Plantar, Plantar Fasciitis, Chronic, Fasciitis, Plantar, Chronic, Plasma, Platelet-Rich, Platelet Rich Plasma”. Taking PubMed's search as an example, the literature search strategies are shown in Table 1.

**Table 1:** Search strategy used in PubMed database.

Order	Search items
#1	((Plasma, Platelet-Rich[Title/Abstract]) OR Platelet Rich Plasma[Title/Abstract]))
#2	((((((((((((Plantar Fasciitis[Title/Abstract]) OR Policeman's Heel[Title/Abstract]) OR Heel, Policeman's[Title/Abstract]) OR Heels, Policeman's[Title/Abstract]) OR Policeman Heel[Title/Abstract]) OR Policeman's Heels[Title/Abstract]) OR Policemans Heel[Title/Abstract]) OR Heel Spur Syndrome[Title/Abstract]) OR Chronic Plantar Fasciitis[Title/Abstract]) OR Fasciitis, Chronic Plantar[Title/Abstract]) OR Plantar Fasciitis, Chronic[Title/Abstract]) OR Fasciitis, Plantar, Chronic[Title/Abstract])
#3	((((randomized controlled trial[Publication Type]OR randomized[Title/Abstract]OR placebo[Title/Abstract])))
#4	#1 AND #2 AND #3

### 2.3 Inclusion criteria

#### 2.3.1 Types of studies

This study only considered clinical RCTs of GCs and PRP in the treatment of PF.

#### 2.3.2 Types of participants

Diagnosed PF, meeting clinical diagnosis criteria, did not accept GC or PRP treatment recently.

#### 2.3.3 Types of interventions

The intervention measures were PRP and GC local injection into metatarsal fascia, PRP and GC were respectively in treatment group and control group.

#### 2.3.4 Outcome measures

The primary outcomes were evaluated by Visual Analogue Scale and Ankle Hindfoot Scale.

### 2.4 Exclusion criteria

(1) Lack of PRP or adopted GC as intervention;
(2) Adopted different basic therapy;
(3) Final indicator does not include Visual Analogue Scale or Ankle Hindfoot Scale;
(4) Repetitive contents;
(5) Non-clinical RCT; non-human trial;
(6) Baseline date (age etc) between 2 group shows a statistical difference;
(7) Being evaluated to low quality research by Jadad scale.

### 2.5 Selection of studies and data extraction

**Figure 1:**
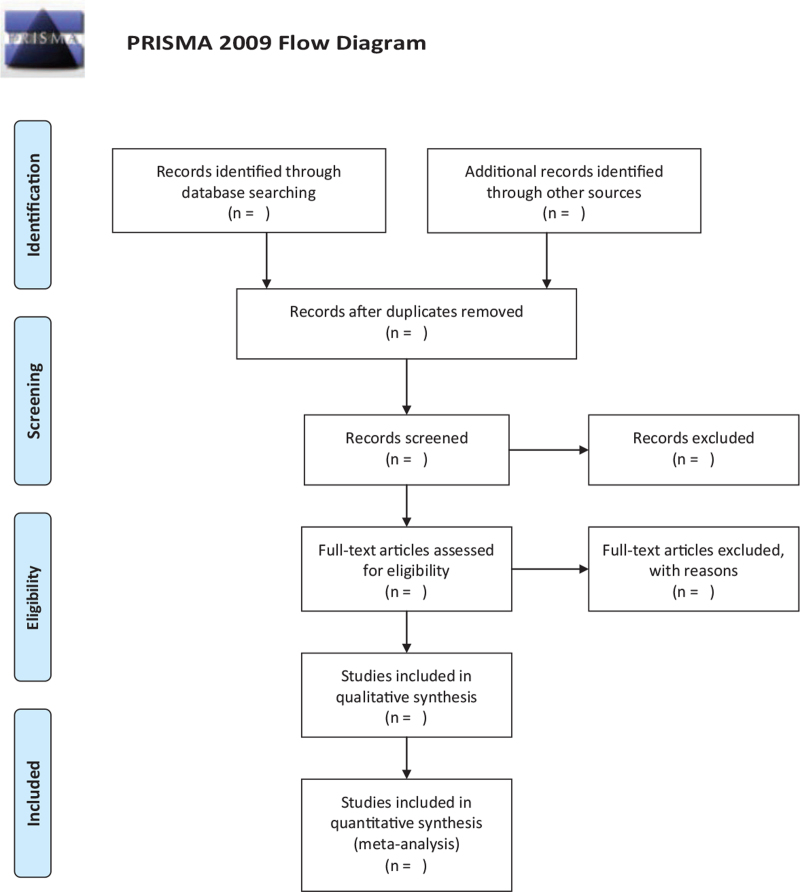
Flow chart of study selection.

First of all, eliminate repetitive contents been searched, conducted by EndNote X9. Then, exclude contents against standard after reading their title and abstract. Third, exclude contents against standard after reading the whole contents. Fourth, extraction of literature data. These data contain method of research design, interventions, methodology, baseline date (age etc) between 2 group, final indicator, follow-up and missing situation, etc. What have been mentioned above were conducted by 2 independent evaluators, and any differences that were difficult to determine could be solved by the third independent evaluator. The selection process will be shown in Figure 1 with the PRISMA flow diagram.

Next, 2 independent evaluators will extract data from qualified literature according to a pre-designed data extraction table. The extracted content included author's name, year of publication, article title, sample size, gender and age of participants, diagnostic criteria, information about intervention and control groups, intervention measures, follow-up time, outcome indicators, and outcomes. Any disagreement will be resolved through consultation with a third independent evaluator.

### 2.6 Risk of bias assessment

The “risk of bias assessment” tool recommended in Cochrane System Assessment Manual 5.0 was used to evaluate the included clinical randomized controlled studies.^[19]^ According to Cochrane Handbook 5.0, ① stochastic method; ② allocation concealment; ③ adopt blinding to volunteers and researchers; ④ adopt blinding to evaluator; ⑤ the completeness of research data; ⑥ selective reporting study outcomes; ⑦ other bias. To decide whether it is low bias risk, bias risk unsure or lack of information. What have been mentioned above were conducted by 2 independent evaluators, and any differences that were difficult to determine could be solved by the third independent evaluator.

### 2.7 Date synthesis and statistical methods

Using Stata12.0 software and RevMan 5.3 software to do statistical treatment, outcome indexes performed in odds ratio. For the dichotomous outcomes, we will use the relative risk to measure the treatment effect, and for the continuous outcomes, we will use standard mean difference to analyze the effect. Both calculating 95% confidence intervals.

### 2.8 Assessment of heterogeneity

Adopting I^2^ test (test level α = 0.05) to assess statistical heterogeneity of the studies. When I^2^ < 50%, it indicates that there is a small statistical heterogeneity or no significant statistical heterogeneity between studies, using a fixed effect model; when I^2^ > 50%, it indicates that the data of studies exist considerable heterogeneity, applying random effects models to do combined analysis. When the heterogeneity is greater, the source of heterogeneity needs to be further analyzed.

### 2.9 Assessment of reporting biases

Galbraith radial plot and Egger test were used to evaluate the potential reporting bias of the inclusion study.

### 2.10 Subgroup analysis and sensitivity analysis

If there is a large heterogeneity between the studies, we will conduct a subgroup analysis to investigate the differences in age and sex, measure of intervention, etc. And we will also use Stata12.0 software for sensitivity analysis to assess the robustness of the study conclusions. If the results showed no qualitative change in the combined effect, the results are stable.

### 2.11 Literature quality evaluation

Using Jaded trial to evaluate clinical RCT's score. The total mark was 5, take scores below (include) 2 as low quality research, scores more than 2 as high quality research. Evaluation standard was: ① randomized blind: The total mark was 2, if there mentioned “randomized blind” and its synonymy gets 1 mark, any specific describe of achieving method about randomized blind add another 1 mark; ② withdraw and loss of follow up: total mark was 1, with explanation about withdraw situation gets 1 mark.

### 2.12 Ethics and dissemination

This study does not collect the personal information of clinical trial participants, so no ethical approval is required. The result of this research will provide reliable evidence-based medical evidence for the long-term efficacy of PRP and GC in treating PF, and the research will be published in peer-reviewed journals.

## 3 Discussion

PF is the most common cause of chronic heel pain in adults, and its pathological manifestations are local aseptic inflammation and fascia rupture. There are many clinical treatment methods, among which GC and PRP are the common treatment methods for PF. However, there is still a lack of systematic evaluation of the clinical efficacy of PRP and GC in the treatment of PF. In this study, a systematic review and meta-analysis were conducted for the comparison of the long-term efficacy of the 2 drugs in the treatment of PF, hoping to provide evidence-based medical evidence for the treatment of PF and guide clinical decision-making.

## Author contributions

**Conceptualization:** Rongrong Ge, Jun Wu.

**Data curation:** Bangmin Wang, Jiawang Jiang, Qianan Cao.

**Formal analysis:** You Zeng, Yunfeng Liu.

**Methodology:** Jiawang Jiang, Bangmin Wang.

**Software:** Rongrong Ge, Qianan Cao.

**Supervision:** Rongrong Ge, Bangmin Wang.

**Writing – original draft:** Rongrong Ge, Bangmin Wang, Shuying Chen, Jiawang Jiang.

**Writing – review & editing:** Rongrong Ge, Shuying Chen, Jiawang Jiang, You Zeng.

## References

[1] Goff JD Crawford R. Diagnosis and treatment of plantar fasciitis. Am Fam Physician 2011;84:676–82. 21916393

[2] Bai WB Liu LR Lu J. Progress in diagnosis and treatment of plantar fasciitis. Chin J Bone Joint Surg 2021;14:805–10.

[3] Monto RR. Platelet-rich plasma and plantar fasciitis. Sports Med Arthrosc Rev 2013;21:220–4. 24212370 10.1097/JSA.0b013e318297fa8d

[4] Wearing SC Smeathers JE Urry SR Hennig EM Hills AP. The pathomechanics of plantar fasciitis. Sports Med 2006;36:585–611. 16796396 10.2165/00007256-200636070-00004

[5] Beeson P. Plantar fasciopathy: revisiting the risk factors. Foot Ankle Surg 2014;20:160–5. 25103701 10.1016/j.fas.2014.03.003

[6] Thomas JL Christensen JC Kravitz SR. The diagnosis and treatment of heel pain: a clinical practice guideline–revision 2010. J Foot Ankle Surg 2010;49:s1–9. 20439021 10.1053/j.jfas.2010.01.001

[7] Cole C Seto CK Gazewood JD. Plantar fasciitis: evidence-based review of diagnosis and therapy. Am Fam Physician 2005;72:2237–42. 16342847

[8] League AC. Current concepts review: plantar fasciitis. Foot Ankle Int 2008;29:358–66. 18348838 10.3113/FAI.2008.0358

[9] Riddle DL Schappert SM. Volume of ambulatory care visits and patterns of care for patients diagnosed with plantar fasciitis: a national study of medical doctors. Foot Ankle Int 2004;25:303–10. 15134610 10.1177/107110070402500505

[10] Scher DL Belmont PJ Bear R Mountcastle SB Orr JD Owens BD. The incidence of plantar fasciitis in the United States military. J Bone Joint Surg Am 2009;91:2867–72. 19952249 10.2106/JBJS.I.00257

[11] Xiao J Peng JG Yang YF Wen JM. Adult calcaneodynia: an interpretation of consensus on diagnosis and management of plantar fasciitis. Electron J Foot Ankle Surg 2020;7:01–5.

[12] Wei FY Qu F Wang XJ Zhang JZ. Therapeutic effectiveness of platelet-rich plasma injection on chronic plantar fasciitis. Chin J Front Med Sci 2019;11:43–6.

[13] Bicer M Hocaoglu E Aksoy S İnci E Aktaş I. Assessment of the efficacy of extracorporeal shockwave therapy for plantar fasciitis with magnetic resonance imaging findings. J Am Podiatr Med Assoc 2018;108:100–5. 29634309 10.7547/15-106

[14] Cheng H Lu WP Gao XZ. Effect of ultrasound-guided platelet-rich plasma injection on plantar fasciitis. J Clin Anesthesiol 2018;34:1072–5.

[15] Acevedo JI Beskin JL. Complications of plantar fascia rupture associated with corticosteroid injection. Foot Ankle Int 1998;19:91–7. 9498581 10.1177/107110079801900207

[16] Boswell SG Cole BJ Sundman EA Karas V Fortier LA. Platelet-rich plasma: a milieu of bioactive factors. Arthroscopy 2012;28:429–39. 22284405 10.1016/j.arthro.2011.10.018

[17] Almeida AM Demange MK Sobrado MF Rodrigues MB Pedrinelli A Hernandez AJ. Patellar tendon healing with platelet-rich plasma: a prospective randomized controlled trial. Am J Sports Med 2012;40:1282–8. 22472272 10.1177/0363546512441344

[18] Shamseer L Moher D Clarke M. Preferred reporting items for systematic review and meta-analysis protocols (PRISMA-P) 2015: elaboration and explanation. BMJ 2015;350:g7647. 25555855 10.1136/bmj.g7647

[19] Higgins JP Altman DG Gøtzsche PC. The Cochrane Collaboration's tool for assessing risk of bias in randomised trials. BMJ 2011;343:d5928. 22008217 10.1136/bmj.d5928 PMC3196245

